# Health risk analysis of micro-and nanoplastic exposure via the microbiota-gut-brain axis

**DOI:** 10.3389/fimmu.2026.1762813

**Published:** 2026-02-18

**Authors:** Xuan Wang, Haoming Yu, Jihong Li, Shengyu Han, Yuhua Chi

**Affiliations:** 1 Department of General Medicine, Affiliated Hospital of Shandong Second Medical University, Weifang, Shandong, China; 2 College of Clinical Medicine, Shandong Second Medical University, Weifang, Shandong, China

**Keywords:** gut microbiota, health risks, intestinal barrier dysfunction, micro- and nanoplastics, microbiota-gut-brain axis

## Abstract

As global plastic pollution continues to intensify, micro- and nanoplastics have emerged as novel environmental pollutants threatening human health. These particles enter the human body through dietary ingestion, inhalation, and skin contact,accumulating within the gastrointestinal tract. Their disruption of the intestinal microbiota has become a recent research focus. Studies indicate that micro- and nanoplastics can interfere with the normal mechanisms of the microbiota-gut-brain axis via neural, immune, and endocrine pathways, thereby inducing or exacerbating diseases. This paper reviews the exposure pathways and intestinal accumulation characteristics of micro- and nanoplastics in humans, the composition of normal gut microbiota and their metabolic products, elucidates the functional pathways of the microbiota-gut-brain axis and the potential impacts of microplastics upon entering the human body, and summarises the current research status, limitations, and future prospects.

## 1 Introduction

In recent years, environmental pollution caused by plastic products has become a focal point of global concern. Plastic production has surged from 1.5 million tonnes in 1950 to 430.9 million tonnes in 2024, with projections indicating continued growth by 2050 (1). Approximately 79% of plastic waste enters the environment through landfilling, incineration, and other means. After prolonged exposure to multiple factors, including abiotic (physical and chemical) and biotic (animal and microbial) influences, it degrades into smaller fragments collectively termed micro- and nano plastics (MNPs). Plastic fragments smaller than 5 mm in diameter are defined as microplastics (MPs), while those smaller than 1 μm are typically classified as nanoplastics (NPs) (2, 3). MNPs are now widely present in drinking water, soil, air and various foodstuffs, and have been detected in human placenta and faeces, among other sites (1, 4). MNPs contamination has emerged as an additional global challenge following climate change and heavy metal pollution.Early studies hypothesised that the primary accumulation sites for MNPs were the liver, followed by the kidneys, and finally the intestines. However, recent research indicates that the principal accumulation sites for MNPs in the human body are the gastrointestinal tract and lymph nodes, particularly the small intestine, followed by the liver and spleen (5). Research indicates that only 20–45% of MNPs within the digestive tract can penetrate the intestinal mucosal barrier to enter the circulatory system. Particles smaller than 150 μm may traverse the intestinal epithelium, while larger plastic fragments remain trapped within the intestinal mucus. There, they persistently interact with the gut microbiota, intestinal epithelial cells, and immune cells.Dysbiosis of the gut microbiota is closely associated with the onset of various gastrointestinal disorders such as inflammatory bowel disease (IBD) and irritable bowel syndrome (IBS), metabolic disorders, and cardiovascular diseases (6–9). MNPs can disrupt the intestinal microecology through neural, immune, and metabolic pathways via the Microbiota-gut-brain axis(MGBA) (10, 11).

Although recent studies have confirmed that MNPs influence gut microbiota composition and diversity, existing research remains limited. Most investigations utilise experimental animal models, with only sparse data addressing the effects of MNPs exposure on human gut microbiota. Current understanding of the toxic effects of MNPs remains limited, with insufficient data on dose-response relationships. Predictions suggest direct effects occur only at extremely high doses. Research on the impact of MNPs exposure on the MGBA is particularly scarce, and the underlying mechanisms remain poorly elucidated (12, 13). This paper analyses the mechanisms by which MNPs influence microbial homeostasis via MGBA, examining aspects such as human exposure to MNPs, gut microbiota composition, and metabolic products. It further explores the health risks associated with dysbiosis, thereby providing theoretical framework for research into MNPs-related diseases and the development of prevention and treatment strategies.

## 2 Exposure routes of microplastics in the human body

MNPs are categorised by origin into primary microplastics, manufactured directly in minute sizes as raw materials added to cosmetics, facial cleansers and similar products, and secondary microplastics generated by the fragmentation of macroplastics in the environment (12). The chemical types of MNPs frequently detected in the environment are primarily polyethylene (PE), polypropylene (PP), polystyrene (PS), and polyethylene terephthalate (PET). Among these, PS constitutes the most common MNPs component in food packaging and disposable takeaway containers, and PS is also one of the most extensively studied MNPs (14, 15). MNPs are ubiquitous in the environment, with their presence detectable in soil ecosystems, surface water, coastal sediments, and sandy beaches; even rain and snow contain substantial quantities of MNPs. It is estimated that humans ingest an average of 0.1–5 grams of MNPs weekly through various exposure pathways, including dietary intake, inhalation, and skin contact, with dietary exposure being the predominant route (16). The largest source of weekly MNPs intake per capita globally is drinking water, with MNPs detected in bottled water, tap water, surface water, and groundwater. Among foods, shellfish, beer, and salt exhibit the highest MNPs concentrations (17). In 2021, Schwab detected at least one type of MNPs exceeding the corresponding limit of quantification in human faecal samples. PE and PET were detected in over 60% of samples, while PS was found in 12 out of 15 participants’ faecal specimens. This study suggests MNPs may accumulate within the gastrointestinal tract (18).

The intestine, as the primary organ of accumulation for MNPs, exhibits a particle size-dependent penetration of MNPs through its physiological barrier. In 2022, Marine et al. observed in a sea urchin intestinal model that particles ≥10 μm predominantly remained in intestinal fluid and were excreted via faeces. High-density polyethylene(HDPE)MNPs measuring 1-5 μm can penetrate the intestinal wall through intercellular gaps in the epithelium or via phagocytosis, entering the portal circulation and depositing in other organs. However, some MNPs persistently remain in the gut, continuously disrupting the intestinal microbiota (19).

## 3 Gut microbiota and their key metabolites

### 3.1 Gut microbiota and their metabolites

The complex community of millions of microorganisms within the human colon constitutes the gut microbiota, whose metabolic activities are crucial for maintaining host homeostasis. The predominant bacterial phyla comprise Firmicutes, Bacteroidetes, Actinobacteria, Proteobacteria, and Verrucomicrobia (20). The gut microbiota plays a vital role in the host’s normal physiological functions: (1) Regarding human metabolism, Firmicutes, Bacteroidetes, and certain anaerobic gut microbes metabolise ingested food into SCFAs, which exert beneficial effects upon entering the colon. Furthermore, the gut microbiota synthesises numerous vitamins beneficial to the human body and neurochemicals that influence the peripheral and central nervous systems (such as gamma-aminobutyric acid). Additionally, the gut microbiota participates in the synthesis of bile acids, cholesterol, and conjugated fatty acids (21). (2) The gut microbiota colonises the intestinal surface, producing diverse antimicrobial substances that prevent pathogenic microbial invasion. It also generates metabolites such as SCFAs, which serve as a vital energy source for intestinal epithelial cells, thereby strengthening the protective function of the intestinal mucosal barrier (7). (3) SCFAs produced by the gut microbiota influence blood-brain barrier (BBB) integrity by increasing tight junction protein expression. Enhanced BBB integrity prevents harmful metabolites from entering brain tissue (22). Compounds such as lipopolysaccharides (LPS) produced by the gut microbiota exert immune functions by stimulating immune cells (microglia) to release cytokines. These cytokines can cross the blood-brain barrier, activate neurons, alter neural function, and consequently lead to changes in mood and behaviour.

#### 3.1.1 SCFAs

SCFAs refer to organic monocarboxylic acids with carbon chain lengths of fewer than six carbon atoms. Primarily composed of acetate, propionate, and butyrate, they are produced by gut microbiota fermenting dietary fibre and other substances. SCFAs perform functions including providing energy to epithelial cells, alleviating systemic inflammation, strengthening the intestinal mucosal barrier, and maintaining colonic homeostasis (23–25). SCFAs play a pivotal role in the MGBA, influencing the brain through multiple mechanisms (26, 27). SCFAs can induce vagal signaling to activate various neurons within the central nervous system; however, further research is required to identify the specific neuronal pathways activated. SCFAs can cross the BBB, exerting direct effects on the brain (28). However, butyrate distribution in the brain is minimal; a study tracking radiolabelled butyrate in primates found that less than 0.006% of administered butyrate reached the brain within 5 minutes post-administration. Further investigation is required to determine whether such minute quantities of gut-derived SCFAs can influence the brain (29). Existing evidence supports SCFAs’ role in modulating BBB permeability: increased permeability was observed in germ-free mice, whereas re-colonisation with complex microbiota restored BBB integrity in these adult mice (30). SCFAs can reduce microglial activation and pro-inflammatory cytokine secretion, alter the integrity of the central nervous system’s blood-brain barrier, and consequently influence the central nervous system and microglial maturation.Butyrate reduces microglial activation and pro-inflammatory cytokine secretion, inhibiting lipopolysaccharide- induced pro-inflammatory modifications. Supplementing the diets of germ-free mice with SCFAs stimulates defective microglial maturation (31–34). SCFAs play a role in maintaining central nervous system homeostasis, accumulating in the hypothalamus to activate the hypothalamic-pituitary-adrenal (HPA) axis while also signaling to enteroendocrine cells. Beyond this, the hippocampus and striatum are similarly susceptible to SCFA influence. One study demonstrated that supplementing drinking water with major SCFAs ameliorated alterations in the HPA axis, intestinal permeability, and anhedonia induced by chronic psychosocial stress in mice (35).

#### 3.1.2 Gamma-aminobutyric acid

Glutamate and Gamma-aminobutyric acid (GABA) are two crucial neurotransmitters in the human central nervous system, with glutamate serving as the primary excitatory neurotransmitter and GABA functioning as the principal inhibitory neurotransmitter (36). Gut bacteria can convert glutamate into GABA via glutamate decarboxylase, with Escherichia coli and Lactobacillus species are also capable of synthesising GABA (37, 38). GABA exerts its effects by inhibiting the production of pro-inflammatory cytokines, promoting the generation of immunomodulatory molecules, regulating the inhibitory-excitatory balance essential for brain function, and influencing neuropeptide secretion by enteric neuropeptide fibres (39).

#### 3.1.3 Dopamine and noradrenaline

Bacteria within the gut, such as Escherichia coli and Bacillus species, can produce dopamine (DA) and noradrenaline (NE), thereby regulating multiple central and peripheral nervous system functions (39). DA is implicated in regulating behaviour, cognition, emotion, motor function, memory, and learning. It modulates effector immune cell function, activates T cells to produce cytokines, reduces the suppressive activity and adhesion/migration capacity of regulatory T cells(Tregs) (associated with neurodegeneration), regulates nitric oxide synthesis, and influences microglial migration (40–42). Dysfunction of the central dopaminergic system and its associated pathways has been linked to Parkinson’s disease and schizophrenia (43). NE plays a role in attention, long-term memory, and behavioural flexibility. Within the brain, noradrenaline(NE) modulates excitability and inter-neuronal responses, while also exerting neuroprotective effects by inhibiting inflammatory gene transcription and enhancing the production of brain-derived neurotrophic factor by microglia and astrocytes (44, 45). Dysfunction of the noradrenergic system is associated with Parkinson’s disease, Alzheimer’s disease, and anxiety disorders (46).

#### 3.1.4 5-hydroxytryptamine

Enterochromaffin cells(ECs) in the gut constitute the primary source of serotonin, responsible for synthesising 95% of the body’s 5-hydroxytryptamine (5-HT). Dysbiosis of the gut microbiota can disrupt the gastrointestinal serotonergic system (24). For instance, Bacteroides polymorpha within the gut microbiota can activate chromaffin cells to induce 5-HT production (47). Although 5-HT cannot cross the blood-brain barrier, it can activate 5-HT3 receptors on afferent fibres of the vagus nerve. This transmits information to the nucleus of the solitary tract, which then propagates signals to the amygdala, locus coeruleus, and other brain regions, thereby influencing cerebral signaling activity, participating in numerous central nervous system disorders (e.g., depression, anxiety, schizophrenia, Parkinson’s disease) and peripheral organ dysfunctions (e.g., gastrointestinal diseases, arrhythmias) (48, 49). The gut microbiota, through its metabolic products, can modulate the immune system, maintain the intestinal mucosal barrier, and influence human physiological functions. This also provides clues to the mechanisms by which MNPs affect microbial community composition, thereby inducing or exacerbating disease. Nevertheless, our understanding of the connections between 5-HT, MGBA, and disease remains nascent, necessitating further research to substantiate these interrelationships (50).

The gut microbiota participates in gut-brain signaling through the aforementioned metabolites, and this signaling extends far beyond unidirectional transmission: these metabolites actively engage in a dynamic bidirectional regulatory network that integrates neural, immune, and endocrine pathways. The following sections will elucidate the framework of MGBA’s bidirectional regulatory mechanisms, detailing how bottom-up (gut-to-brain) and top-down (brain-to-gut) signals coordinate systemic homeostasis.

## 4 Bidirectional regulation of the MGBA

The MGBA builds upon the gut-brain axis (GBA) by incorporating the regulatory influence of gut microbiota. It retains the original components of the GBA axis while introducing the microbiota and its metabolites, thereby forming a more complex communication axis. This represents the natural evolution and expansion of the GBA axis within the contemporary research landscape of the microbiome. The intestinal barrier is a complex structure primarily composed of the mucus layer, epithelial cell layer, and mucosal basement membrane, encompassing mechanical, chemical, immune, and biological barriers (51, 52). The gut microbiota exerts bottom-up regulation on the central nervous system primarily through neuroimmune and neuroendocrine mechanisms, frequently involving the vagus nerve. Immune cells within the gut recognize microorganisms via pattern recognition receptors. Under normal conditions, Toll like receptors (TLR2/4), through Myeloid Differentiation Primary Response 88 (MyD88), can modulate the expression of Interleukin-10(IL-10) and Transforming growth factor β(TGF-β), thereby maintaining the M2 phenotype of macrophages. This reduces intestinal ischemia/reperfusion injury and inflammatory responses, thus sustaining intestinal homeostasis (53–55). When confronted with pressure or stress, the gut microbiota can more effectively constrain the adrenocorticotropic hormone (ACTH) and cortisol produced by the HPA axis (56). Secreted propionic acid binds to G protein-coupled receptor 43(GPR43), stimulating the release of colonic tissue-derived peptide (PYY) and glucagon-like peptide-1 (GLP-1). This subsequently inhibits the activity of agouti-related protein(AgRP)-associated neurons, thereby reducing food intake (57). Furthermore, butyrate can upregulate uncoupling protein 2 (UCP2) to reduce mitochondrial reactive oxygen species (ROS), while UCP2 promotes M2 phenotype polarization of microglia, thereby suppressing inflammatory responses (58, 59). SCFAs can also directly cross the blood-brain barrier to exert effects in the brain, for instance, acetate directly increases proopiomelanocortin (POMC) expression while decreasing AgRP neuronal activity in the hypothalamus, leading to a sharp reduction in food intake (60). Butyrate-derived ketone bodies rapidly cross the blood-brain barrier, increasing antidiuretic hormone (ADH) secretion and influencing water-salt metabolism (61).

**Figure 1:**
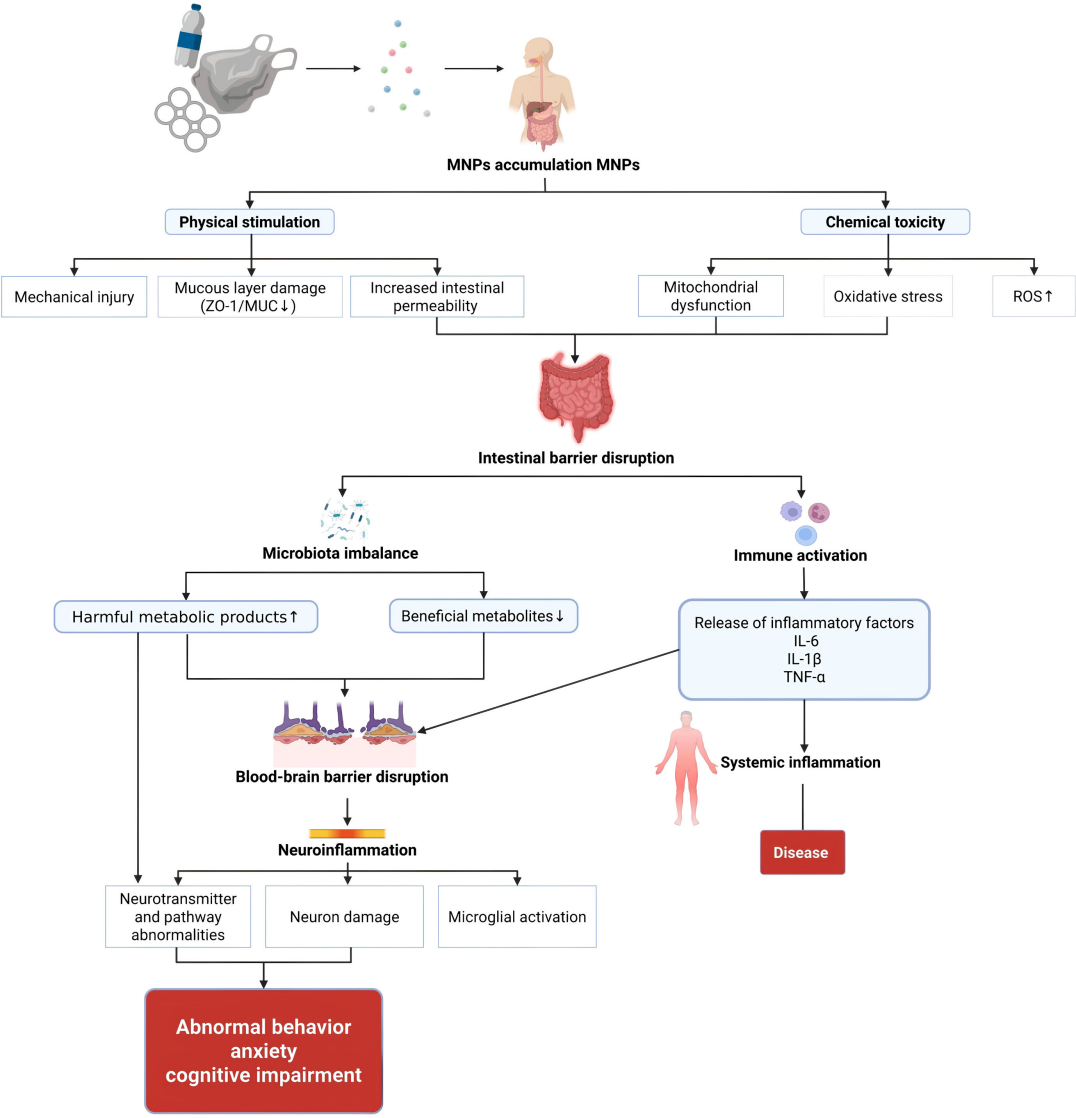
Schematic diagram of the bidirectional regulatory mechanism of MGBA.

The gut-brain axis involves intricate communication through neural, immune, and endocrine pathways. Microbial metabolites and 5-HT from enteroendocrine cells stimulate vagal afferents, transmitting signals to the central nervous system. The central nervous system subsequently modulates gut function via vagal efferents; the microbiota and its metabolites regulate immune cells. This interaction leads to the release of cytokines that can signal to the brain to induce neuroinflammation. Systemic inflammation may also compromise the intestinal barrier; microbiota-derived SCFAs influence the HPA axis and hormone secretion, thereby transmitting signals to the brain. The hypothalamic-pituitary-adrenal axis, activated by stress or inflammation, regulates the gut microbiota and barrier function via glucocorticoid feedback.Conversely, prolonged elevation of cortisol levels alters gut microbiota composition and increases gastrointestinal permeability, thereby negatively impacting gut microbiota function. In summary, the gut-brain axis establishes bidirectional regulation through endocrine, immune, and neural pathways. However, MGBA regulation is susceptible to interference from both internal and external environmental factors. In recent years, the persistent accumulation of ubiquitous novel environmental pollutants-MNPs-within the human gastrointestinal tract has posed unprecedented challenges to the functional integrity of MGBA. These particles can disrupt one or multiple links in the aforementioned bidirectional regulatory pathways through various mechanisms, including direct physical interactions, induction of oxidative stress, and triggering of chronic inflammation. The following sections will systematically elucidate the specific mechanisms by which MNPs interfere with MGBA (See **Figure 1**).

## 5 Interference of MNPs with MGBA

### 5.1 Disrupting gut microbiota homeostasis

#### 5.1.1 Mechanical damage and mucosal injury

The uptake of MNPs in the gut depends on their particle size and occurs via multiple mechanisms. These include microvillus-mediated phagocytosis through microvilli cells, endocytosis through intestinal cells such as clathrin-coated pit-mediated endocytosis, permeation, and paracellular pathways. Due to the absence of enzymes capable of degrading plastics, particles smaller than 150 μm can penetrate the intestinal mucus layer, while those larger than 150 μm remain attached to its surface, directly contacting the apical portion of epithelial cells (62, 63). Particles smaller than 100 μm can traverse the intestinal barrier to reach other barriers (64). Among these, nanoscale particles can embed within the hydrophobic core of the lipid bilayer, forming a disentangled network of monomer chains. This network alters cell membrane function by affecting the bilayer structure, ultimately leading to cell death (65). Upon entering the gut, these foreign particles act as irritants, causing mechanical damage to the digestive tract by abrasion. This disrupts the intestinal mucosa, leading to increased intestinal permeability (the so-called ‘leaky gut’ phenomenon) and triggering additional inflammation. Furthermore, as poorly degradable polymeric organic compounds, MNPs are difficult for the gut to absorb. These particles occupy the intestinal lumen, impeding normal nutrient absorption, disrupting the normal nutritional supply to the gut microbiota, and impairing the function of the symbiotic microbial community (66, 67).

Polystyrene nanoplastics (PS-NPs) within MNPs may accumulate in the gut, altering the expression of gut microRNA-501-3p and microRNA-700-5p. This disruption compromises the expression of tight junction protein ZO-1 and mucins (such as MUC-13), weakening the mucus layer, increasing intestinal permeability, and diminishing the protective function of the intestinal barrier (68). Furthermore, electrostatic attraction may also induce damage to the intestinal mucosal barrier. Positively charged MNPs particles can interact with negatively charged mucins via electrostatic attraction, reducing mucin diffusion rates, impeding normal mucus hydration and dispersion, and increasing the viscosity of the mucin network. This facilitates particle retention, leading to a thinner intestinal mucus layer and compromised intestinal mucosal barrier integrity (69). Compromised intestinal mucosal barriers and increased retained particles heighten the probability of MNPs penetrating the intestinal barrier. These particles interact with various cells, activating Nod-like receptor protein 3(NLRP3) inflammasomes and inducing inflammatory responses (70).

#### 5.1.2 Oxidative stress

MNPs can generate ROS within cells through multiple mechanisms. These particles themselves can produce ROS, and their chemically reactive surfaces can catalyse Fenton reactions to generate ROS (71). Furthermore, MNPs may adsorb metals, organic pollutants, and pesticides onto their surfaces. These adsorbed substances can induce ROS generation through diverse chemical reactions (72). For instance, polycyclic aromatic hydrocarbons adsorbed onto MNPs can generate ROS via photoreactions under ultraviolet radiation (71). Photooxidation or ultraviolet radiation can generate free radicals on the MP/NP surface by removing hydrogen atoms from macromolecular chains or adding hydrogen atoms to unsaturated carbon chain groups. These free radicals within the polymer chains can react with atmospheric oxygen to produce polymeric peroxy radicals (73, 74). The activation of NADPH oxidase (NOX) represents one key mechanism. In mouse studies, it was discovered that MNPs can activate multiple pathways, including TLR4 and the aryl hydrocarbon receptor (AhR). Upon activation of these pathways, NOX generates superoxide anions, which can be converted into other ROS (71, 75). MNPs can promote ROS production by disrupting mitochondrial function, leading to excessive ROS generation within the electron transport chain (ETC) (76, 77). Additionally, MNPs induce cytochrome c translocation, activate caspases, and trigger apoptosis (78). MNPs may suppress the production of antioxidant enzyme transcription factors or reduce antioxidant enzyme activity, thereby inhibiting ROS metabolism. This increases mitochondrial membrane potential, elevates mitochondrial permeability, and accelerates ROS transfer from mitochondria to the cytoplasm. The accumulation of ROS within the cell causes oxidative damage to cellular components (79).

#### 5.1.3 Gut microbiota dysbiosis

In mouse models, exposure to MNPs (such as PE, PET, PP, PS, and Polyvinyl chloride(PVC) microplastics) has been shown to disrupt the composition of the gut microbiota (80). The Firmicutes phylum significantly increased while the Bacteroidetes phylum decreased in the polypropylene-exposed group, with a rapid rise in the Firmicutes/Bacteroidetes ratio-a marker strongly associated with obesity. Conversely, the relative abundance of the Human-inhibiting colitis (HIC) genus, which suppresses colitis in humans, markedly declined in the polystyrene group (81, 82). Even among particles of the same type, the pattern of microbial imbalance varies across different anatomical sites. PS exposure significantly reduced the relative abundance of Lactobacillus grunwaldii and Romboutsia ilealis within the intestinal microbiota of carp (83). Differences in relative abundance following PET exposure varied across distinct colonic regions. In the ascending colon, levels of the Firmicutes and Desulfobacterota increased, while the proportion of Bacteroidetes decreased. In the transverse colon, the proportions of the Synergistetes, Proteobacteria, and Desulfobacterota rose, whereas the relative abundance of Bacteroidetes declined markedly to below 10%. In the descending colon, Synergistetes levels increased, whilst the relative abundance of the Bacteroidetes phylum, after a significant decline exceeding 15% within the first 24 hours, subsequently stabilised (84).

Experimental findings exhibit species variability and are influenced by exposure duration and MNPs particle characteristics, rendering them incapable of reflecting genuine human physiological changes. Clinical trials concerning MNPs exposure remain insufficient. Comparative analysis of gut microbiota between high- and low-exposure cohorts suggests that elevated exposure may increase the abundance of disease-associated microbes while reducing beneficial microbial richness, providing direct evidence of MNPs’ impact on human gut microbiota (85).

### 5.2 Activation of gut immunity inducing inflammation

#### 5.2.1 Activation of gut immune cells and release of inflammatory mediators

Existing research has found that after entering the body, MNPs can activate innate immune cells (macrophages, dendritic cells, natural killer cells, etc.) and adaptive immune cells (T cells, B cells, etc.), promoting the release of inflammatory cytokines.Through the NF-κB and MAPK pathways, NLRP3 inflammasome assembly and activation, and Toll-like receptors (TLRs), MNPs synergistically induce the release of interleukin-1β (IL-1β), interleukin-6 (IL-6), and tumour necrosis factor-α (TNF-α) (86–90). For instance, animal studies demonstrate that following MNPs exposure, macrophages phagocytose these foreign particles, stimulating polarization towards either pro-inflammatory M1 or anti-inflammatory M2 phenotypes. Smaller particles exhibit more pronounced MAPK pathway activation, and the resulting increase in reactive oxygen species (ROS) further biases macrophage polarization towards the M1 phenotype (87). Macrophage phagocytosis of MNPs elevates ROS levels, activates the MAPK/NF-κB pathway, and enhances IL-6 and TNF-α secretion (88). Concurrently, MNPs promote NLRP3 inflammasome assembly and activation, releasing interleukin-1β (IL-1β) (89). Through these mechanisms, local inflammatory responses develop within the body.

#### 5.2.2 Breaching the blood-brain barrier induces neuroinflammation

Peripheral inflammatory mediators may enter the central nervous system via the vagus nerve or humoral pathways. Peripheral signals (inflammatory mediators) in the peritoneal cavity directly activate the vagus nerve to transmit signals to the central nervous system by binding to receptors at the nerve endings of vagal fibres (91). For instance, animal studies reveal that vagotomy attenuates the expression of pro-inflammatory factors in the brain following peripheral TNF-α exposure (92). Within the bloodstream, these inflammatory mediators primarily signal to the CNS via the circumventricular organs (CVOs), by crossing the BBB, or by activating vascular cells at the BBB, thereby influencing cerebral inflammatory responses. These pathways may also act in combination. Firstly, due to the absence of a barrier function in CVOs, inflammatory cytokines in the bloodstream can enter the brain via these structures (93). Secondly, inflammatory cytokines can promote degradation of tight junction proteins in BBB endothelial cells, thereby increasing permeability (94). Concurrently, systemic inflammation induced by dysbiosis can upregulate adhesion molecules on BBB endothelial cells, exacerbating immune cell infiltration into the brain (95, 96). Furthermore, systemic perivascular macrophages or inflammatory mediators may directly activate signaling pathways in vascular cells, influencing intracerebral inflammatory responses (97). Following entry of inflammatory mediators into the nervous system, microglia transition from a quiescent state to a pro-inflammatory activated state, producing neurotoxic pro-inflammatory mediators that induce neuronal death and promote neuroinflammation (98). Moreover, IL-1α and TNF-α secreted by microglia can induce astrocytes to produce neurotoxic factors, thereby exacerbating neuroinflammation.The NLRP3 inflammasome has also been implicated in neuroinflammation, with chronic colitis potentially mediating neuroinflammation through NLRP3 inflammasome activation; however, the precise mechanisms remain unclear (98, 99). Naturally, the HPA axis also participates in immune responses. It monitors alterations in gut microbiota composition and function; when dysbiosis occurs, HPA axis activation induces inflammatory signaling pathways, releasing TNF-α, IL-6 and other factors that compromise blood-brain barrier integrity and promote the progression of cerebral disorders. Furthermore, HPA-axis-induced inflammation influences glucocorticoid secretion, modulating gut function and pro-inflammatory factor production. This activates intestinal immune cells, such as Th17 and NK cells, which invade the brain to initiate or exacerbate neuroinflammation (100). Concurrently, inflammation mediates mast cells (MC) activation via corticotropin-releasing hormone (CRH), further elevating intestinal permeability. This establishes a brain-gut feedback loop that intensifies the inflammatory response (101).

### 5.3 Disruption of neurotransmitter synthesis and metabolism

Beyond altering microbial composition, MNPs-induced dysbiosis in animal models profoundly changes microbial metabolites, affecting neurotransmitter synthesis and metabolism. Exposure to MNPs impacts the synthesis and metabolism of multiple neurotransmitters, primarily affecting SCFA production in the intestine (102). For instance, reduced Bacteroidetes phylum abundance may diminish SCFA production, adversely affecting gut health (103). Furthermore, studies indicate that the type of MNPs influences these alterations: polyethylene increases acetate levels while decreasing propionate and butyrate, whereas polyethylene terephthalate (PET) reduces butyrate and acetate levels (104, 105). Under normal conditions, SCFAs maintain intestinal barrier function, interact with G protein-coupled receptors, and regulate T cells to reduce inflammatory responses. When their production diminishes, these functions weaken, leaving the gut vulnerable to disease (106). MNPs may also influence host cell metabolism through oxidative stress, leading to abnormal elevations in brain DA and GABA levels, manifesting as anxiety and behavioural abnormalities. Animal studies reveal that ultraviolet irradiation accelerates MNPs ageing. When danio rerio are exposed to environmentally relevant concentrations (0.1–100 μg/L) of virgin polystyrene (V-PS) and photoaged polystyrene(P-PS)(10 μm) for 120 hpf (hours post-fertilization), neurotransmitter levels-including 5-HT,GABA, DA, and acetylcholinesterase (AChE) -significantly increase, markedly reducing juvenile swimming velocity and even inducing epileptic-like behaviour (49). In adult zebrafish, exposure to medium-high concentrations of PE microplastics also induced anxiety-like behavior, but metabolic changes were not examined (50). In mouse experiments, PS(5.0–5.9 μm) exposure was found to impair learning and memory by inducing oxidative stress and reducing acetylcholine levels (107). Animal data cannot replicate human responses, and corresponding clinical trials remain lacking. However, recent three-dimensional models simulating human brain development indicate that prolonged MNPs exposure diminishes neuronal viability (108).

### 5.4 Disruption of neurological gene expression and signaling pathways

MNPs may also interfere with the expression of neuro-related genes and disrupt normal signaling pathways. In fish MNPs exposure experiments, genes associated with neural activity and neurotransmitter receptors within the brain transcriptome exhibited upregulation of 5-Hydroxytryptamine Receptor 3(HTR3), Sphingosine-1-Phosphate Receptor 4(S1PR4), Cholinergic Receptor Nicotinic Gamma(CHRNG), Cholinergic Receptor Nicotinic Gamma(PLG), cAMP Responsive Element Binding Protein 3(CREB3), Cholinergic Receptor Muscarinic 4(CHRM4), and Solute Carrier Family 6 Member 9, SLC6A9/GlyT1(GLYT), while Arrestin 3(ARR3), Hypocretin Receptor 2(HCRTR2), POMC, and Adrenergic Receptor Alpha-1B(ADRA1B) genes were downregulated. Kyoto Encyclopedia of Genes and Genomes(KEGG) pathway enrichment analysis indicated that MNPs may interact with the neuroactive ligand-receptor interaction pathway, Serotonergic synaptic pathways and dopaminergic synaptic pathways. GLYT were upregulated, while ARR3, HCRTR2, POMC, and ADRA1B were downregulated. KEGG pathway enrichment analysis indicated MNPs’ association with neuroactive ligand-receptor interactions, serotonergic synaptic pathways, and dopaminergic synaptic pathways—all linked to synaptic transmission and emotional expression (109, 110).

Although these conclusions can be drawn from animal studies, clinical trial evidence remains lacking, and the precise mechanism of action is currently unclear. In summary, MNPs interfere with MGBA through a multi-pathway synergistic effect. The potential mechanism by which MNPs induce health risks through interference with MGBA is elucidated by describing their initial role in disrupting intestinal homeostasis.

**Figure 2:**
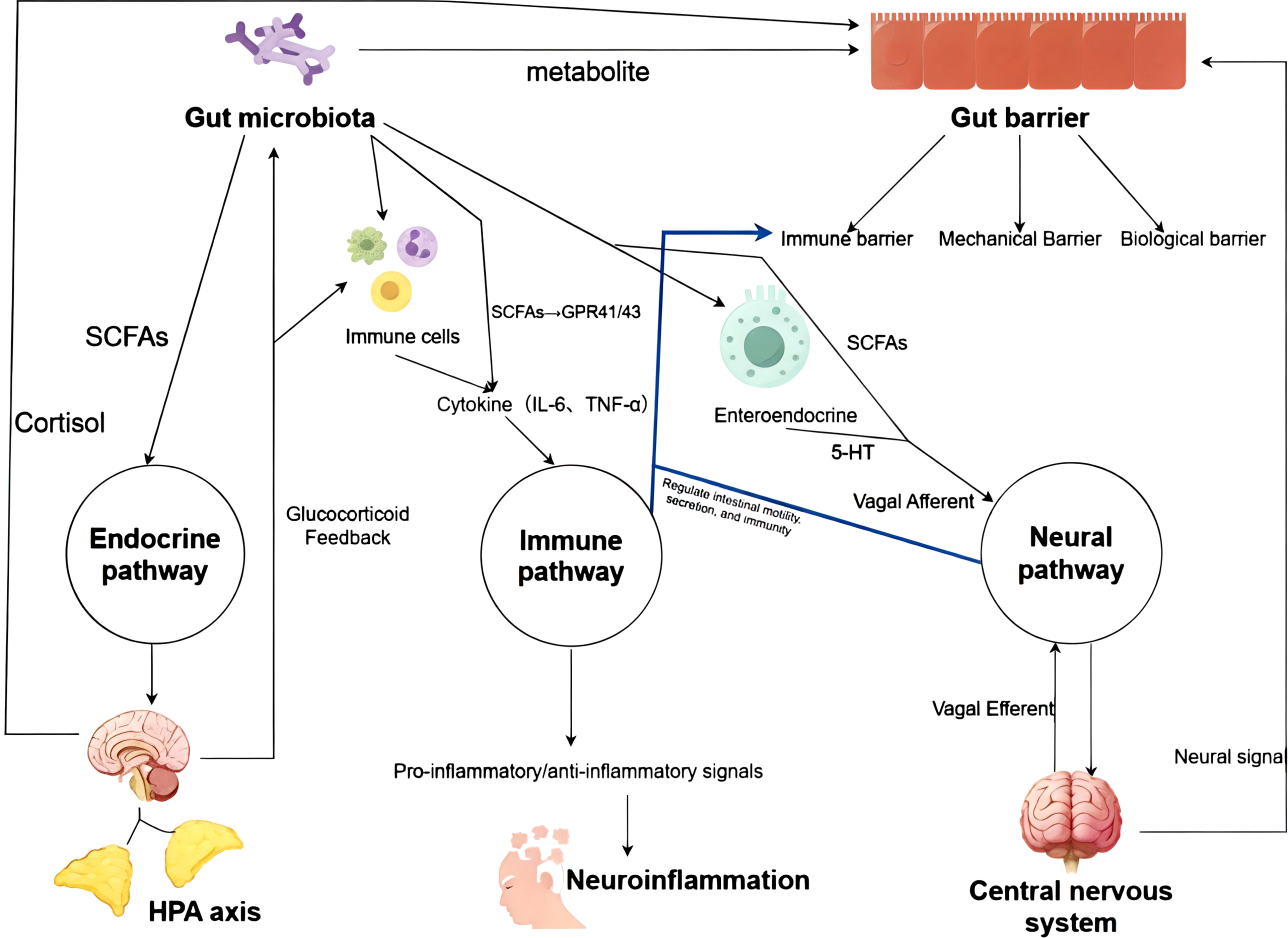
MNPs induce health risks through MGBA.

MNPs disrupt MGBA through multi-pathway synergistic effects, initially destabilizing intestinal homeostasis and ultimately affecting distant organ function via neural, immune, and endocrine pathways as shown in **Figure 2**. However, these insights primarily stem from animal models and *in vitro* studies. While they provide important mechanistic hypotheses, direct clinical evidence remains scarce, and the precise relevance of these pathways to human pathophysiology requires further investigation.

### 5.5 Summary of experimental data

To clearly present the core experimental evidence demonstrating how MNPs affect the body via MGBA in the aforementioned studies, and to facilitate rapid reference to different research models, plastic types, plastic doses, and corresponding experimental results, **Table 1** systematically collates and summarises relevant literature reviewed earlier including animal models, *in vitro* experiments, and clinical studies.

**Table 1:** Summary of experimental evidence on MNPs affecting the MGBA.

Experimental model	Plastic type	Experimental results	Reference
Animal model			
Danio rerio	Exposed to environmentally relevant concentrations (0.1–100 μg/L) of V-PS and P-PS (10 μm) for 120 hpf	P-PS induced more severe neurotoxicity than V-PS, characterized by decreased locomotor behavior, altered antioxidant enzyme activities and MDA content, elevated neurotransmitter levels (DA, 5-HT, GABA, ACh), and significantly altered expression of neurotransmission- and oxidative stress-related genes.	(49)
Adult zebrafish	Exposed for 96 h to five single-size (10-22, 45-53, 90-106, 212-250, and 500-600 μm, 2 mg/L) and three mixed-size (11, 110, and 1,100 particles/L) PE microplastics.	Anxiety-like behavior was observed at medium-high concentrations.	(50)
The C57BL/6 male mice	Mice were orally administered PS-NPs (100 nm) at a concentration of 2×10¹¹ particles/mL four times per week for 12 weeks.	Intestinal NP accumulation was accompanied by increased body weight, altered expression of miR-501-3p and miR-700-5p, impaired ZO-1 and MUC-13 expression leading to increased intestinal permeability, and gut microbiota dysbiosis characterized by increased Ruminococcaceae and decreased Lactobacillus.	(68)
Kunming mice	Mice were orally administered PE, PET, PP, PS, and PVC microplastics (150-300 μm) at 20 mg/mL by gavage (0.2 mL/day) for 7 days.	While physiological indicators showed no significant changes, colonic tissues exhibited pathological damage (most severe in the PS group with inflammatory cells accounting for 21.53%), and gut microbiota structure was altered, characterized by an elevated Firmicutes/Bacteroidetes ratio in PE, PET, and PP groups and significantly reduced Alistipes abundance in the PS group.	(82)
Carp	PS	Decreased abundance of Lactococcus garvieae and Romboutsia ilealis.	(83)
Mice	PS(5.0–5.9 μm) administered orally at 0.01, 0.1, and 1 mg/day for 3 weeks	can induce oxidative stress, reduce the production of acetylcholine and inhibit the phosphorylation of CREB, resulting in the impairment of learning and memory abilities in mice.	(107)
Discus fish	Continuous 96-hour exposure to microfibers (900 µm, fiber, MFs) or nanoplastics (~88 nm, bead, NPs) at concentrations of 0, 20, and 200 µg/L.	MFs inhibited growth performance; NPs impaired swimming and predatory capacity; brain-gut neurotransmitter imbalance; gut microbiota structural alterations (Proteobacteria, Clostridia, and Fusobacteriia shifts); brain transcriptome neural pathway enrichment (neurobehavioral toxicity).	(109)
*in vitro* model			
Mouse mononuclear macrophage leukemia cells (RAW264.7)	PS of two sizes (50 nm and 500 nm) at concentrations of 10 and 50 μg/mL were applied for 24–48 hours of exposure.	Macrophages rapidly internalized nanoplastics; at 50 μg/mL, this promoted pro-inflammatory M1-type macrophage polarization, increasing CD86, iNOS, and TNF-α expression while decreasing CD206, IL-10, and Arg-1 expression.	(86)
Mouse mononuclear macrophage leukemia cells (RAW264.7)	Macrophages were exposed to surface-functionalized polystyrene nanoplastics (PS, PS-COOH, PS-NH_2_, 80 nm) and microplastic PS (5 μm) at concentrations ranging from 10–1000 μg/mL for 24 hours.	PS-COOH exhibited the highest intracellular accumulation and strongest induction of ROS and apoptosis; PS-COOH and PS-NH_2_ significantly activated the MAPK/NF-κB pathway at a low concentration of 10 μg/mL, promoting IL-6 and TNF-α release; and the inflammatory effects of PS-COOH could be suppressed by the ROS inhibitor NAC.	(88)
*In vitro* simulated human colon digestion	PET at 166 mg per dose (simulating human daily intake) was subjected to oral-gastric-small intestinal digestion, followed by 72 hours of colonic fermentation.	PET surfaces developed organic deposits and biofilm-like structures; Raman spectroscopy revealed structural amorphization of PET; microbial α-diversity decreased; Bacteroidetes declined while Firmicutes, Proteobacteria, and Desulfobacterota increased; and opportunistic pathogens (Escherichia/Shigella, Bilophila) proliferated.	(84)
Clinical research			
Human cohort (High vs. Low exposure)	Environmental microplastic mixtures (mainly PU) with long-term exposure (>3 years,>20 h/day)	The levels of microplastics in the nasal cavity and intestines significantly increased; pathogenic bacteria such as Klebsiella and Helicobacter rose in the nasal microbiota, while beneficial bacteria like Bacteroides declined; in the gut microbiota, Bifidobacterium, Streptococcus, and Sphingomonas decreased, while Ruminococcus Torquesgroup, Dorea, and Fusobacterium increased.	(85)
IBD patients and healthy individuals	MNPs in feces	The concentration of MNPs in the feces of IBD patients is higher and positively correlated with disease severity.	(64)

## 5 Health risks associated with MNPs

### 5.1 MNPs and gastrointestinal disorders

Following exposure to MNPs, not only can gastrointestinal disturbances occur, but there is also a high likelihood that they may induce gastrointestinal pathologies such as IBD and IBS by affecting the MGBA. MNPs concentrations in the faeces of IBD patients were significantly higher than in healthy individuals, with a markedly greater number of MNPs ≤50 micrometres observed. Concurrently, research revealed that PET exhibited greater relative abundance in IBD patients, whilst MNPs concentrations showed a positive correlation with IBD severity (64). Mechanistically, MNPs induce intestinal immune cells to promote the release of inflammatory cytokines, thereby triggering acute inflammation. Prolonged presence of these cytokines may evolve into chronic inflammation, inducing IBD. This process is also associated with MNPs’ disruption of the intestinal mucosal barrier (111). Given the current scarcity and high heterogeneity of studies, it remains inconclusive to determine the precise pattern of inflammatory cytokine alterations during MNPs-induced or exacerbated IBD. MNPs may also influence IBS progression via the gut-brain axis. Research indicates that the onset of IBS may be associated with the gut microbiota, inflammation, and emotional factors. Dysbiosis of the microbiota triggers immune activation, potentially promoting intestinal inflammation in some IBS patients. The cause of low-grade systemic inflammation in IBS is intestinal permeability, specifically compromised intestinal barrier integrity, allowing intestinal fluids (such as immune cells and microbiota) to enter the circulation and induce inflammation. Furthermore, the onset of IBS is frequently associated with negative emotions (101). Although research linking IBS and MNPs remains scarce, MNPs can induce dysbiosis, inflammatory responses, and negative emotions such as depression via MGBA. This mechanism could potentially trigger or exacerbate IBS, offering a direction for future investigations.

### 5.2 MNPs and other systemic diseases

**Figure 3:**
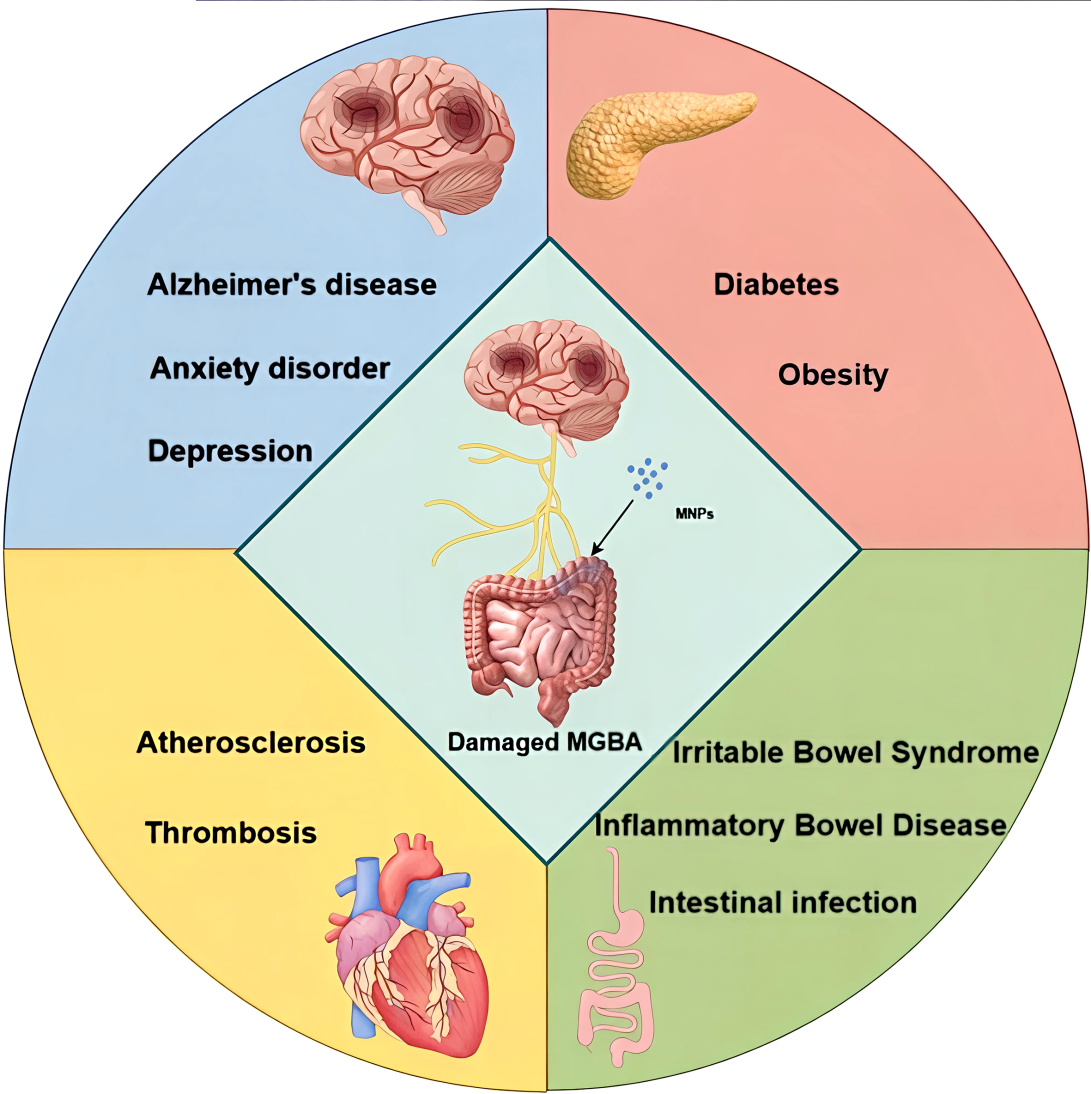
MNPs and multiple system injuries.

Beyond gastrointestinal disorders, MNPs may contribute to the onset or progression of various systemic diseases by disrupting gut microbiota homeostasis. Regarding metabolic conditions, the gut microbiota exhibits close associations with obesity and diabetes. Research indicates that in diabetic patients, the proliferation of the phyla Proteobacteria, Bacteroidetes, and Firmicutes within the gut microbiota exceeds that observed in healthy individuals. In the context of cardiovascular disease, gut microbiota can trigger secondary cardiovascular conditions such as diabetes and obesity. Furthermore, dysbiosis-induced intestinal barrier dysfunction, alongside the accumulation of lipopolysaccharides and toxins, accelerates atherosclerosis and thrombus formation. Neurological disorders including stroke, glioma, Alzheimer’s disease, and depression are also associated with gut microbiota within the MGBA (112). Consequently, the health risks posed by MNPs are not confined to a single system but may involve simultaneous impairment across multiple systems (see **Figure 3**). This figure visually illustrates the multiple organ systems potentially affected simultaneously or sequentially following MNPs exposure through pathways such as MGBA. These include the digestive system (e.g., IBD, IBS), metabolic system (e.g., obesity, diabetes), cardiovascular system (e.g., atherosclerosis), and nervous system (e.g., neurodegenerative diseases, mood disorders), underscoring the extensive scope of their health implications.

## 6 Conclusion

In recent years, environmental pollution caused by plastic products has become a matter of global concern. These materials enter the human body through ingestion, respiration, and skin contact, accumulating in the gastrointestinal tract. They subsequently disrupt the normal mechanisms of the MGBA via neural, immune, and endocrine pathways. This paper systematically examines the pathways of the MGBA by analysing human exposure routes to MNPs, their accumulation characteristics in the gut, and their effects on normal gut microbiota composition and metabolic products. It demonstrates how MNPs entering the human body can induce or exacerbate diseases, posing health risks, thereby providing theoretical reference for research and prevention strategies concerning MNPs-related illnesses.

However, it must be acknowledged that current research in this field has significant limitations. The vast majority of evidence originates from animal models such as rodents and fish, as well as *in vitro* experiments. While these animal models are indispensable for elucidating the mechanisms by which MNPs disrupt the MGBA, the exposure scenarios in these experiments-including dose, duration, and particle type—often differ from the complex and variable human environment. Consequently, conclusions drawn from animal studies cannot be directly equated with human health risks. Direct evidence clearly establishing a causal link between specific MNPs exposure levels and alterations in human gut microbiota homeostasis remains extremely limited. A few clinical studies observing correlations between fecal MNPs levels and diseases like IBD are constrained by small sample sizes and the difficulty of accurately quantifying lifetime exposure doses, preventing definitive causal conclusions. Furthermore, research methodologies remain inconsistent. Existing studies vary in particle size, concentration, and exposure duration of MNPs, lacking standardized dose protocols and toxicity assessment frameworks. This significantly impairs comparability between research outcomes and hinders the establishment of safe exposure thresholds. Finally, mechanistic research requires integration and deepening. Current research predominantly focuses on single pathways (e.g., inflammatory mediators, SCFA alterations) and lacks systematic integration of interactions across multiple systems such as the nervous, immune, and endocrine systems. A comprehensive mechanistic map has yet to be established. Furthermore, different polymer types (e.g., PS, PE, PET) and their aged or surface-modified states exhibit significant variations in their effects on the microbiota and host. Existing studies primarily analyze single polymers, lacking systematic comparative research and mechanistic explanations.

## 7 Future outlook

To address the aforementioned knowledge gaps and advance the field toward precise risk assessment and effective interventions, future efforts should focus on the following areas:

### 7.1 Conduct prospective population monitoring studies

Utilize large-scale samples combined with mass spectrometry imaging to precisely quantify MNPs concentrations in humans across different life stages and exposure scenarios. Conduct dynamic analyses linking these concentrations to microbiota, metabolites, and clinical health risks.

### 7.2 Strengthen research on particle-specific and combined exposure effects

Systematically compare the combined toxicity profiles of MNPs with varying chemical compositions, particle sizes, and surface characteristics, alongside coexisting environmental pollutants, to more accurately reflect the complexity of real-world exposure scenarios.

### 7.3 Explore intervention and mitigation strategies

Based on mechanistic research, investigate the feasibility of reducing MNPs bioavailability or mitigating associated intestinal and neurological damage through dietary fiber, prebiotics/probiotics, specific adsorbent materials, and other approaches.

In summary, the potential health risks posed by MNPs via MGBA represent a complex emerging topic spanning environmental science, microbiology, neuroscience, and toxicology. Despite significant challenges, multidisciplinary collaboration, the adoption of research models more closely aligned with human physiology, and rigorous epidemiological investigations hold promise for unraveling the truth about this environmental health risk. This will provide a scientific basis for developing evidence-based public health policies and personal protective guidelines.
